# Association of socioeconomic position with sensory impairment among Chinese population: a nationally representative cohort and Mendelian randomization study

**DOI:** 10.3389/fpubh.2024.1371825

**Published:** 2024-04-18

**Authors:** Jin Wei, Yifan Zhou, KaiweiSa Abuduxukuer, Jialong Dong, Chuchu Wang, Wenming Shi, Jianfeng Luo, Qing Peng, Yi Song

**Affiliations:** ^1^Department of Opthalmology, Shanghai Municipal Hospital of Traditional Chinese Medicine, Shanghai University of Traditional Chinese Medicine, Shanghai, China; ^2^Department of Ophthalmology, Shanghai Tenth People’s Hospital, School of Medicine, Tongji University, Shanghai, China; ^3^Department of Biostatistics, School of Public Health, Fudan University, Shanghai, China; ^4^School of Public Health, Fudan University, Shanghai, China; ^5^Shanghai Collaborative Innovation Center of Industrial Transformation of Hospital TCM Preparation, Shanghai, China

**Keywords:** Mendelian randomization study, visual impairment, socioeconomic position, hearing impairment, sensory impairment

## Abstract

**Aims:**

To investigate the association between socioeconomic position (SEP) and sensory impairments (SIs).

**Methods:**

We used data from the China Health and Retirement Longitudinal Study (CHARLS) (2015). Logistic regressions estimated the odds ratio for associations of SEP with SIs. In addition, Mendelian randomization (MR) analysis was conducted to assess the causal relationship between them with the inverse variance weighting (IVW) estimator. MR-Egger, simple median, weighted median, maximum likelihood, and robust adjusted profile score were employed for sensitivity analyses.

**Results:**

In the observational survey, we enrolled 19,690 individuals aged 45 and above. SEP was negatively associated with SIs. Adjusted odds of vision impairment were higher for illiterate (1.50; 95%CI: 1.19, 1.91), less than elementary school diploma (1.76; 95%CI: 1.39, 2.25), middle school diploma (1.53; 95%CI: 1.21, 1.93) and lower income (all *p* < 0.001). The odds of hearing impairment were significantly higher for people with less than a high school diploma than those with a college degree or higher diploma, for agricultural workers than non-agricultural workers, and for people in low-income families (*p* < 0.01). The MR analysis also showed that occupation was associated with HI (1.04, 95%CI: 1.01, 1.09, *p* < 0.05) using IVW.

**Conclusion:**

We found that both observational and causal evidence supports the theory that SEP can result in SIs and that timely discovery, targeted management, and education can prevent SIs among middle-aged and older adults.

## Introduction

Sensory impairments (SIs), including vision impairment (VI) and hearing impairment (HI), are chronic functional impairments associated with aging (1). In the world, more than one-third of all people over 65 suffer from HI, and approximately 80% of people suffering from HI or more than mild VI are over 50 (2). There is a severe problem of aging society in China. The incidence of SI there has grown faster than in other G20 countries (3). From 19.65 million in 1999 to 45.92 million in 2019, Chinese adults with moderate VI have doubled. It is estimated that 68.62% of older adults with a mean age of 69 had moderate or severe hearing loss, according to a hearing survey conducted in China in 2019 (4). The prevalence of VI in urban and rural areas was 0.58 and 1.15%, respectively, with significant statistical differences (5). About 16 ~ 24% of HI can be attributed to occupation-related factors (6). SIs are essential public health problems in China, with substantial regional and occupational variations in prevalence (7).

The relationship between health and socioeconomic position (SEP) has been observed in many countries (8). An individual’s SEP is determined by their education level, occupation tier, personal income and wealth, and household income and wealth. Indeed, the lower the SEP of an individual, the poorer their health across global society (9). Increasing pieces of evidence from Western countries have indicated that low SEP significantly increases the risk of onset of SIs (10–15). Only a few observational studies have been conducted on SIs in China (16–19), and they identified that SIs are linked to poverty and geographic location. Since existing literature consists only of cross-sectional and regional studies with no indication of directionality (16), the relationship between SEP and SIs is not thoroughly investigated.

It remains to be determined if SEP plays a role in the differential prevalence of SIs in different provinces and even countries. Due to logistical issues and costs, conducting randomized controlled trials can take time and effort. In addition, traditional observational studies can make it difficult to quantify causal effects due to residual confounding and reverse causality (20). Mendelian randomization (MR) is an alternative means of determining causality. MR employs genetic variants to explore association and causation, which can overcome some of the limitations of observational studies and establish a causal relationship between SEP and SI risk (21). The genetic variants used as instrumental variables (IV) confirm the credibility of the MR study (22). The genetic variants, also named single nucleotide polymorphisms (SNPs), were used as IVs for exposures of interest. In this approach, alleles are randomly assigned, so reverse causality and unmeasured confounding factors are less likely to influence the results (23). In general, the results of MR studies are consistent with the results of randomized clinical trials (24). Further, using summary statistics from a genome-wide association study (GWAS), MR designs increase causality inference’s statistical power (25). To our knowledge, no MR Studies have evaluated the association between SEP and SI.

Therefore, considering the limited evidence on this topic among the middle-aged and older Chinese population and the complex relationship between SI and SEP, a national representative dataset from the China Health and Retirement Longitudinal Study (CHARLS) in 2015 was used to expand the currently available literature. MR methods were used to extend observational association to causal association to address previous research limitations and prevention strategies.

## Materials and methods

### Study design and data sources

**Figure 1:**
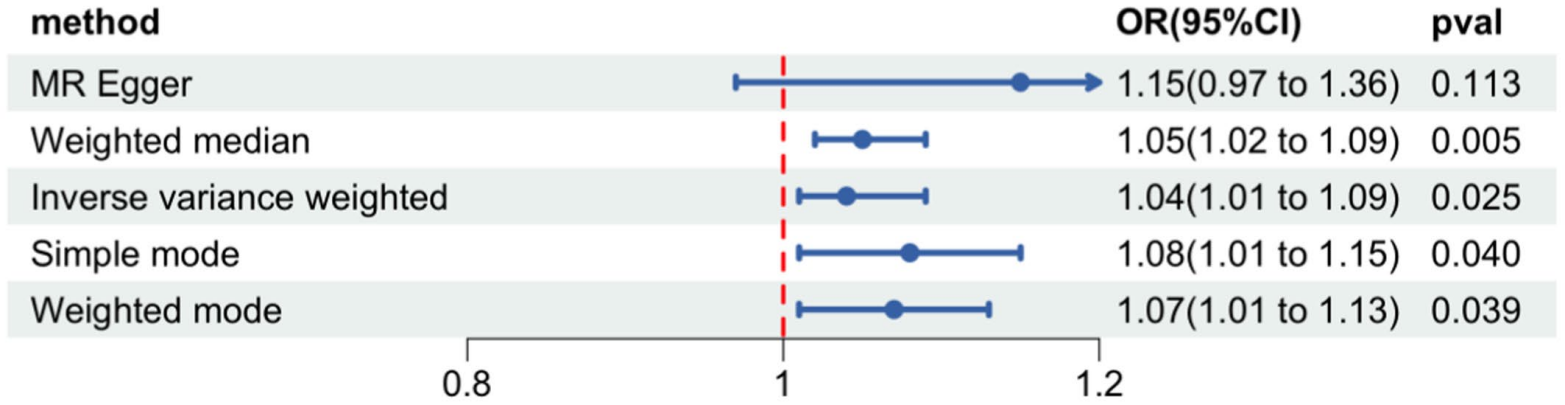
Forest plot of Mendelian randomization for the relationship between occupational attainment and hearing impairment. The random effects inverse-variance weighted (IVW) method was utilized in the main MR analyses. MR-Egger regression, the weighted median, simple mode, and weighted mode were performed as complementary analyses.

Data from the China Health and Retirement Longitudinal Study (CHARLS) were drawn from 28 provinces in mainland China, based on multistage probability sampling and face-to-face interviews. It focuses on socioeconomic status, health, and community activities (26). A baseline survey of approximately 17,000 respondents was conducted between 2011 and 2012, followed by three follow-up interviews in 2013, 2015, and 2018. In this study, we utilized data from the third wave of the 2015 survey, which had a sample size 21,095. After excluding respondents with critical missing information, 19,703 respondents were included in the cross-sectional analysis (Figure 1). The ethics approval number was IRB00001052-11015, and all participants provided written informed consent for CHARLS.

### Sensory impairment

In CHARLS, a single question was used to assess HI: “Is your hearing excellent, very good, good, fair, or poor?” (with a hearing aid if you usually use it and without if you do not).” HI was categorized as having fair or poor responses to this question. Two questions were used to evaluate VI (1): “How good is your vision for seeing things at a distance (with glasses or corrective lenses), like recognizing a friend from across the street?” (2) “How good is your vision for seeing things up close (with glasses or corrective lenses), like reading ordinary newspaper print?” If the respondent reported fair or poor vision (far or near vision), we classified them as VI.

### Socioeconomic position

We use the most traditional indicators of SEP, including occupation status, education level, and annual *per capita* household expenditure, which have proved very useful in describing and evaluating health inequalities (27). The occupation was operationalized as a dichotomous variable. Rather than categorizing people based on their current employment status (given that some people were retired), it was done based on their lifetime profession: People who have exclusively performed agricultural work (without any nonagricultural work for >10 days) throughout their lives were categorized as being involved in agricultural work; Individuals who have worked non-agricultural jobs for more than 10 days (regardless of whether they have worked agricultural work as well) were classified as non-agricultural workers. Education attainment in this study was measured as an interval variable ranging from 1 to 5. The education level is 1 (illiterate), 2 (sishu/home school and below), 3 (elementary school), 4 (middle school), and 5 (high school or above) (28); A higher score was indicative of higher education attainment (29). The net post-tax income for each household was calculated based on the household income (shared by the sample individuals in the same household). Households’ total annual income includes wages, bonus incomes, or pensions from household members, as well as income from agricultural, self-employed activities, public transfer, and other types of transferred sources (such as parents, children, and relatives). We calculated household *per capita* income by dividing total household income by the number of members in the household (28).

### Potential covariates

Age (continuous variable), sex (male or female), marital status, smoking status, drink status, and self-reported history of chronic diseases were selected as covariates. Marital status represented whether respondents lived alone or were accompanied. Respondents who were separated, divorced, widowed, or never married were classified as “living alone,” while those who were married or cohabitated were classified as “living with a partner.” Depending on the category of smoking, participants may be current or former smokers, while the category of drinking indicates how often they drink alcohol: none, less than once a month, or more than once a month.

### Two-sample MR analysis

In the study, 3 phenotypes were grouped into SEP, including occupational attainment, educational attainment (years of education), and total household income (average total household income before tax). The genetic instruments for the 3 phenotypes were obtained from publicly available summary-level data. The education-related and income-related phenotypes were obtained from the IEU OpenGWAS database,^1^ a database of 245,322,865,636 genetic associations from 42,335 GWAS summary datasets, for querying or download. The phenotypes of occupational attainment were obtained from the GWAS Catalog database.^2^ Hence, the final datasets included 248,847 individuals for occupational attainment (9,566,222 SNPs), 461,457 individuals for educational attainment (11,972,619 SNPs), and 397,751 individuals for household income (9,851,867 SNPs) (30). Summary statistics for VI (visual impairment including blindness: binocular or monocular) and HI (age-related hearing impairment) were also obtained from the IEU OpenGWAS database. GWAS datasets obtained in our study included 16,380,453 SNPs for VI from 211,769 individuals (903 cases and 210,866 controls) and 10,858,770 SNPs for HI from 330,759 individuals (31). A valid instrumental variable satisfies three assumptions: (1) relevance assumption: it should be strongly associated with the outcome (sensory impairment); (2) exchangeability assumption: it should not share common causes with the outcome (sensory impairment); and (3) exclusion restriction: it influences the outcome only via its effect on the exposure.

We acquired independent genetic variants strongly associated with SEP (*p* < 5 × 10–8) by clumping SNPs with linkage disequilibrium (LD) *r*^2^ > 0.001 at a window size of 10,000 kb. Following this, SNPs were filtered according to the following procedures: (a) Multiple phenotypic SNPs were discarded to avoid the pleiotropic effect; (b) After extracting the SNPs from the GWAS data for outcomes (VI, HI). Following that, we performed SNP filtering according to a series of steps: (a) SNPs associated with more than one phenotype were discarded, and (b) we extracted SNPs from GWAS datasets (VI, HI). After removing all SNPs associated with outcomes at genome-wide significance. (c) We homogenized exposure-outcome datasets to remove strand-ambiguous SNPs with intermediate allele frequencies (AF > 0.42). (d) The MR-pleiotropy residual sum was utilized to detect and eliminate SNPs with potential pleiotropy at the threshold of *p* < 0.05. No proxy SNPs were used as IVs in MR analysis. The random effects inverse-variance weighted (IVW) method was used to estimate causal effects. We performed MR-Egger regression as complementary analyses, the weighted median, simple mode, and weighted mode. Specific methods can provide valid evidence for different circumstances. For sensitivity analysis, heterogeneity test (Cochran Q test), pleiotropy test (MR-Egger intercept test), and leave-one-out analysis were performed to evaluate the stability of these genetic variants on occupation-related traits.

### Statistical analysis

Baseline data was presented as mean ± standard deviation for continuous variables and number for categorical variables. The differences in baseline characteristics between groups were compared using χ^2^ analysis for categorical variables and analysis of variance or Mann–Whitney U tests for continuous variables. Logistic regression was applied to analyze the association of SEP and SIs. The results are presented as a multivariable-adjusted odds ratio (OR) and 95% confidence interval (CI). Potential covariates included in the multivariable-adjusted model were age, gender, marital status, smoking, drink status, and chronic diseases. Statistical analyses were conducted using SPSS software (V.26.0), and *p* < 0.05 was considered statistically significant. The MR study was conducted in R version 4.1.2 (R Development Core Team, Vienna, Austria) using the “Two-Sample MR” R package version 0.5.6.

## Results

### Observational analysis

Table 1 shows the baseline characteristics of participants in 2015 according to different sensory statuses, including VI and HI. The mean age of participants was 59.16 (SD = 10.21) years, and 52.6% of the participants were female. About 68.4% reported VI, and 63.2% reported HI. HI and VI were more common among older, female, less educated, less income, engaging in non-agricultural work, and living in rural areas. People who have sensory impairments also have less alcohol and tobacco consumption. And they are more likely to suffer from multiple diseases.

**Table 1:** Baseline characteristics of study respondents.

Characteristics	Total	NHI/HI	*p*-value	NVI/VI	*p*-value
Demographic factors					
Number (n, %)	19,690	7,252(36.8)/12,438(63.2)		6,208(31.6)/13,461(68.4)	
Age (M, SD)	59.16(10.21)	57(9.93)/60.42(10.17)	<0.001	57.78(10.58)/59.78(9.96)	<0.001
Gender (n, %)			0.008		<0.001
Male	9,339(47.4)	3,529(48.7)/5,810(46.7)		3,097(49.9)/6,232(46.3)	
Female	10,349(52.6)	3,723(51.3)/6,626(53.3)		3,110(50.1)/7,228(53.7)	
Marital status (n, %)			<0.001		0.367
Married or partnered	17,271(87.7)	6,486(89.4)/10,785(86.7)		5,466(88.1)/11,792(87.6)	
Living alone	2,417(12.3)	766(10.5)/1,651(13.3)		741(11.9)/1,668(12.4)	
Living area (n, %)			<0.001		<0.001
Urban	5,110(26.1)	2,285(31.6)/2,825(22.8)		1827(29.5)/3,281(24.5)	
Rural	14,501(73.6)	4,941(68.4)/9,560(77.2)		4,357(70.5)/10,125(75.5)	
Health factors					
Drinking status (n, %)			<0.001		<0.001
More than once a month	5,273(26.8)	2057(28.4)/3,216(25.9)		1765(28.4)/3,505(26.1)	
Less than once a month	1740(8.8)	690(9.5)/1,050(8.4)		612(9.9)/1,124(8.3)	
Never	12,664(64.4)	4,499(62.1)/8,165(65.7)		3,828(61.7)/8,823(65.6)	
Smoking status (n, %)			0.894		0.943
Current smoker/Ex-smoker	7,989(40.6)	2,938(40.5)/5,051(40.6)		2,517(40.5)/5,465(40.6)	
Non-smoker	11,701(59.4)	4,314(59.5)/7,387(59.4)		3,691(59.5)/7,996(59.4)	
Chronic diseases (n, %)			<0.001		<0.001
No	8,359(42.5)	3,777(52.1)/4,582(36.8)		3,178(51.2)/5,175(38.4)	
One	4,521(23)	1,583(21.8)/2,938(23.6)		1,359(21.9)/3,153(23.4)	
More than one	6,810(34.6)	1892(26.1)/4,918(39.5)		1,671(26.9)/5,133(38.2)	
Socioeconomic position					
Education attainment (n, %)			<0.001		<0.001
Illiterate	6,615(44.9)	1998(39.5)/4,617(47.7)		1848(42.6)/4,749(45.8)	
Less than elementary school	3,266(22.2)	1,017(20.1)/2,249(23.3)		863(19.9)/2,401(23.2)	
Middle school	3,089(15.7)	1,192(23.6)/1897(19.6)		962(22.2)/2,127(20.5)	
High school and vocational school	1,417(7.2)	655(13)/762(7.9)		511(11.8)/906(8.7)	
College and above	341(1.7)	195(3.8)/146(1.5)		149(3.5)/192(1.8)	
Occupation (n, %)			<0.001		<0.001
Agricultural work	9,634(49.1)	3,327(46.1)/6,307(50.9)		2,912(47.1)/6,718(50.1)	
Non-agricultural work	9,990(50.9)	3,894(53.9)/6,096(49.1)		3,274(52.9)/6,699(49.9)	
Income (M, SD)	9039.16(17591.349)	10367.63(21649.6)/8264.6(14663.08)	<0.001	10033.09(22673.08)/8581.23(14646.60)	<0.001

Table 2 shows the regression analyses. It showed that some SEP indicators were associated with VI, including education attainment and income; even after adjustment for all covariates. For example, people with VI were poorer (all *p* < 0.001). The prevalence of VI was higher among illiterate people (OR = 1.923, 95%CI: 1.535–2.409, *p* < 0.001), elementary school graduates (OR = 2.098, 95%CI: 1.664–2.645, *p* < 0.001), middle school graduates (OR = 1.683, 95%CI: 1.337–2.119, *p* < 0.001) and high/vocational school graduates (OR = 1.354, 95%CI: 1.063–1.725, *p* < 0.001). But the association was no longer significant between people with high/vocational school education and college graduates after further adjustment for health covariates. In addition, the prevalence of VI was not statistically obvious among different occupation.

**Table 2:** Logistic regression model was used to describe the correlation between sensory impairment and socioeconomic position.

SEP indicators	VI^a^/OR (95%CI)	VI^b^/OR (95%CI)	VI^c^/OR (95%CI)	HI^a^/OR (95%CI)	HI^b^/OR (95%CI)	HI^c^/OR (95%CI)
Education attainment						
Illiterate	1.923(1.535–2.409)***	1.577(1.246–0.997)***	1.504(1.185–1.908)***	2.782(2.219–3.488)***	2.141(1.689–2.715)***	2.122(1.669–2.698)***
Less than elementary school	2.098(1.664–2.645)***	1.839(1.450–2.333)***	1.764(1.388–2.242)***	2.687(2.131–3.388)***	2.271(1.788–2.883)***	2.252(1.769–2.867)***
Middle school	1.683(1.337–2.119)***	1.570(1.242–1.985)***	1.527(1.205–1.934)***	1.958(1.555–2.465)***	1.859(1.468–2.354)***	1.860(1.465–2.361)***
High school and vocational school	1.354(1.063–1.725)*	1.297(1.016–1.656)*	1.264(0.988–1.617)	1.457(1.144–1.855)**	1.414(1.106–1.807)**	1.410(1.100–1.807)**
College and above	1.000	1.000	1.000	1.000	1.000	1.000
Occupation						
Agricultural work	1.072(0.997–1.152)	1.054(0.972–1.143)	1.056(0.973–1.146)	1.099(1.024–1.179)**	1.099(1.016–1.189)*	1.104(1.021–1.195)*
Non-agricultural work	1.000	1.000	1.000	1.000	1.000	1.000
Income	1.000**	1.000*	1.000*	1.000***	1.000**	1.000**

^a^Model 1 was the crude model. ^b^Model 2 was adjusted for all demographically relevant covariates (age, sex, marital status, living areas). ^c^Model 3 was adjusted for all covariates including demographic and health factors (age, sex, marital status, living areas, chronic diseases, smoking, and drinking status). *p < 0.05, **p < 0.01, ***p < 0.001.

The regression analyses also shows that all SEP indicators were associated with HI, even after being adjusted for multiple confounders (Table 2). HI was more prevalence among people from poor, low-education and agricultural work. For example, agricultural workers had significantly higher odds than non-agricultural workers (OR = 1.099, 95%CI: 1.024–1.179, *p* < 0.01). Odds of HI were significantly higher among people who were high/vocational school graduates (OR = 1.457, 95%CI: 1.144–1.855, *p* < 0.01), middle school graduates (OR = 1.958, 95%CI: 1.555–2.465, *p* < 0.001), those only with elementary school education (OR = 2.687; 95%CI: 2.121–3.388, *p* < 0.001) and those who were illiterate (OR = 2.782; 95%CI: 2.219–3.488, *p* < 0.001) than among college graduates. Respondents from poor households had significantly higher rates of hearing impairment than those from high-income families (*p* < 0.001). Adjustment for all covariates did not alter the results for education attainment, income, and occupation, which remained significant.

### Two-sample MR analysis

Among three phenotypic studies we conducted (e.g., educational attainment, average total household income before tax, occupational attainment), a modest causal relationship was found between occupational attainment and HI (OR = 1.04, 95%CI: 1.01, 1.09, *p* = 0.025) (Figure 1). Supplementary Figures S1, S2 show the scatter plot and the funnel plot. A total of 1,319 SNPs that are strongly associated with occupational attainment at the threshold of statistical significance (*p* < 5*10^−8^) were selected to be IVs. We used a clumping process with the European population to remove bias from LD (*R*^2^ < 0.001, kb = 10,000). Seven SNPs with intermediate allele frequencies palindromes were also removed.

**Figure 2:**
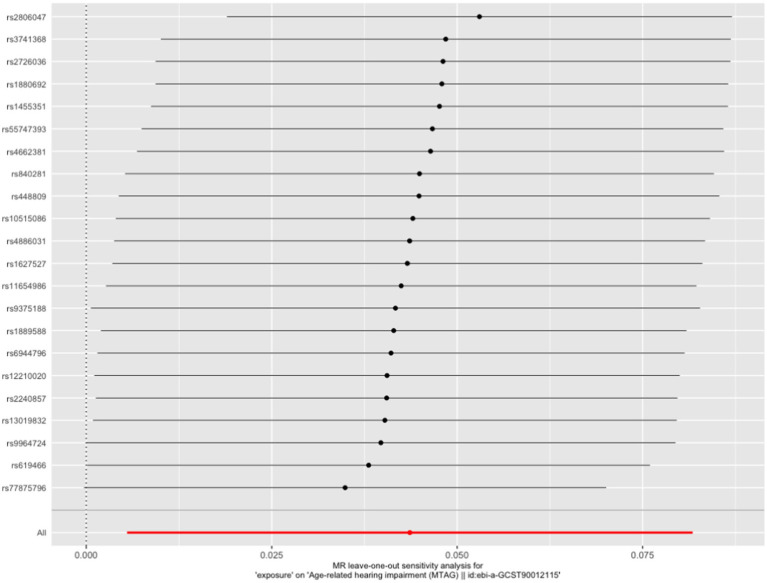
Leave-one-out plot of the effect of occupational attainment on hearing impairment in an MR analysis.

We finally left 29 SNPs for the analysis of occupational attainment and HI (Supplementary Table S1). The Cochran’s Q test for sensitivity analysis demonstrated no significant heterogeneity for the causal effect of occupational attainment on HI (*Q*-pval >0.05). We found no influence of pleiotropy on our results (*p* = 0.26). Finally, the leave-one-out sensitivity analysis verified the stability of the causal inference (Figure 2). The relationship between education and average total household income before tax with HI was not observed, however. No causal relationship was found between the three SEP phenotypes with VI.

## Discussion

As far as we know, this is the first study to provide explicit evidence of the association between SEP and SI in a middle-aged and older Chinese population based on a nationally representative survey and MR analysis. In observational analyses, we found that more than one-half of Chinese individuals who were over 45 years old suffer from SI. HI was associated with household income, educational attainment, and occupation even after being adjusted for all covariates. Household income and educational attainment were consistently related to VI after adjustment. No correlation was found between occupation and VI.

### Educational attainment

Our results showed that educational attainment was inversely associated with VI and HI after adjustment of all related confounders. Lots of cross-sectional studies from different countries indicated a strong, inverse association between education and VI even after adjustment for demographic and behavioral factors (32–34). For example, a study from China showed that a higher educational level was significantly associated with a lower prevalence of VI (35). The negative association was more significant in patients with diabetes from a study in the western Asian region (36). For HI, studies that extracted audiometric measurements from the National Health Interview Surveys 2007 to 2010 waves demonstrated an inverse association between education and HI (10).

A study of the US population found that respondents with more than a high school education have a 70% lower chance of bilateral hearing loss, a 40% lower chance of unilateral hearing loss, and a 50% lower chance of high-frequency hearing loss than respondents without after adjustment (37). The reason for this association may be that the lower the educational attainment, the lower the self-consciousness of VI and HI. Consequently, it is hard to perform timely intervention of SI at an early stage. We found, based on the present study, that the Chinese population, the lower the education level, the greater the risk of SIs.

### Household income

According to our observational analyses, household income was inversely associated with VI and HI. Such associations remained robust even after being adjusted for various confounders. These findings were consistent with two nationally representative studies among American people and Korean adults (10, 38). A strong negative association was found in 190 countries between prevalence rates of VI and national socioeconomic level of development which is measured by gross domestic product *per capita* (39). We also noticed another related study from Japan which reported medium, but not high, household income may be associated with a lower prevalence of HI only in men (40). The prevalence rate of HI is higher in rural areas of China than in economically developed cities (3). However, in another Chinese cross-sectional study of 25,860 working-aged adults (12,804 men and 13,056 women) aged 25–59 years, income was not related to HI (41). The possible reason for such a situation might lay in the fact that the higher-income population would avoid the risk of VI through regular eye examinations, the use of assistive devices such as glasses and portable magnifiers, and surgical procedures (11). On the contrary, interventions such as wearing hearing aids to improve hearing could result in difficulties in manipulating, and poor sense of use which might result in a lower use of hearing aids than expected among the Chinese population (41–43). Therefore, these subjective factors might also need to be taken into account.

### Occupation

We found that occupation has a negative association with HI, which was consistent with other studies among American people and Chinese adults (10, 41, 43). Some studies have mainly attributed this association to occupational noise exposure (45), but others have found that noise exposure was not associated with the 10-year incidence or progression of HI (46). They suggest this may be due to the potential protective effects of variable noise exposure typical in most occupations and the detrimental effects of loud occupational noise exposure. So, longitudinal studies of younger adults may be necessary to elucidate this issue.

We did not find that occupation is related to VI. We firmly believe that our results are correct. The possible explanation is that the mode of Chinese agricultural production has been changing from manual to mechanized production with the rapid economic development and industrialization, which reduced farmers’ exposure to some environmental risks, such as weather, chemicals, plants, and crops that can result in eye injuries (47). Besides, some people are the breadwinners in their families who bear the main economic burden and may have several jobs at once in China. There is a portion of blue-collar workers among non-agricultural workers who are also exposed to some risk factors of VI. The occupational status of the Chinese older adult population may be more complex than in Western countries. There are still few relevant studies in China, and we are the first to explore the association between occupation and VI, waiting for other studies to verify us in the future.

### Mendelian randomization study

The associations found by observational epidemiological studies are insufficient to support causal relationships, as confounding or other forms of bias can cause them (48). Therefore, we conducted MR analysis to comprehend the impact of exposure on outcomes by analyzing genetic variants closely related to exposure. Due to genetic variation being inherited randomly by parents, many factors that might interfere with exposure-outcome relationships cannot affect genetic variation. As genetic variants are generally unaffected by outcomes, reverse causation is not an issue. MR thus offers an opportunity to study the relationship between exposures and outcomes while reducing the potential bias caused by confounding and reverse causation (49). Population-averaged estimates from our genetic analyses suggest that occupation and HI had a causal relationship. Previous studies indicated that harmful occupational exposures, such as high levels of noise, can lead to HI in farmers and construction workers (50, 51). The association between HI and occupation persisted even after adjusting for various confounding factors in the first part of the observation study. The relationship is further strongly demonstrated in MR analysis which needs us to be cogitative. In contrast to some observational studies, we found no causal relationship between other SEP indicators and SIs. Perhaps the disparity is due to the uncontrolled confounding factors in the first part. No causality in the MR may be the low statistical power resulting from the low phenotypic variance from the genetic variants.

### Strengths and limitations

The study has several strengths. First, it is a national study with a large sample size, which means that its findings may be generalizable to the whole country. Second, with data from a national representative investigation in China and MR analyses, this is the first study to explicitly demonstrate an association between SEP and SI among middle-aged and older Chinese populations. Although we carried out comprehensive approaches to profile the relationship between the SEP and SIs, there are still several limitations. Firstly, even though self-reports of SIs have been used in numerous population-based studies (52), a potential bias might result if respondents were misclassified as having SIs or not. It was not assessed whether participants’ assistive devices (e.g., hearing aids, glasses, portable magnifiers) were sufficient to correct their vision or hearing. Despite this, the questionnaire reflects their real-world subjective assessments of their vision or hearing status. Secondly, there are fewer instruments for the MR study, which may introduce weaker instrumental bias and lower statistical power. Lastly, MR results were based on GWAS in participants of European descent, so it is unclear if they apply to other populations as well.

## Conclusion

In summary, the present study provides explicit genetic evidence that occupation has a causal relationship with HI which further substantiates the results of the observational study. Our results have implications for the interpretation of epidemiological causes and possible etiological understanding of SIs, and potential disease prevention strategies.

## Data availability statement

Publicly available datasets were analyzed in this study. This data can be found at: http://charls.pku.edu.cn/.

## Ethics statement

The studies involving humans were approved by the Biomedical Ethics Review Committee of Peking University approved CHARLS, and all participants were required to provide written informed consent. The ethical approval number was IRB00001052-11015. The studies were conducted in accordance with the local legislation and institutional requirements. Written informed consent for participation was not required from the participants or the participants’ legal guardians/next of kin in accordance with the national legislation and institutional requirements.

## Author contributions

JW: Writing – original draft. YZ: Writing – review & editing. KA: Data curation, Writing – review & editing. JD: Methodology, Writing – review & editing. CW: Data curation, Writing – review & editing. WS: Investigation, Writing – review & editing. QP: Conceptualization, Supervision, Writing – review & editing. JL: Conceptualization, Writing – review & editing. YS: Conceptualization, Funding acquisition, Supervision, Writing – review & editing.

## Footnotes

^1^https://gwas.mrcieu.ac.uk/

^2^https://www.ebi.ac.uk/gwas/studies/GCST90102253
