# Supplementary material for: Association of socioeconomic position with sensory impairment among Chinese population: a nationally representative cohort and Mendelian randomization study
Source: Front Public Health. 2024 Apr 18;12:1371825. doi: 10.3389/fpubh.2024.1371825 (PMC11063363; doi:10.3389/fpubh.2024.1371825)
Supplement: Supplementary file 1 [file Data_Sheet_1.PDF]

Supplementary Materials

Supplementary Figure S1 Scatter plot of the effect of occupational attainment on hearing impairment in an MR analysis

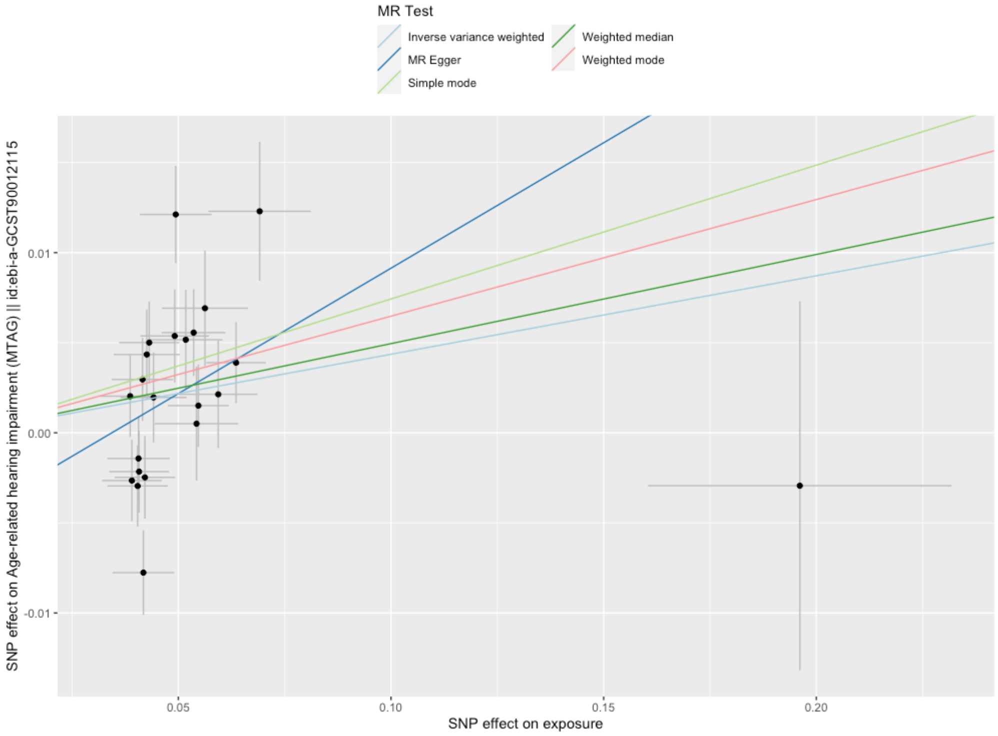

Supplementary Figure S2 Funnel plot of the effect of occupational attainment on hearing impairment in an MR analysis

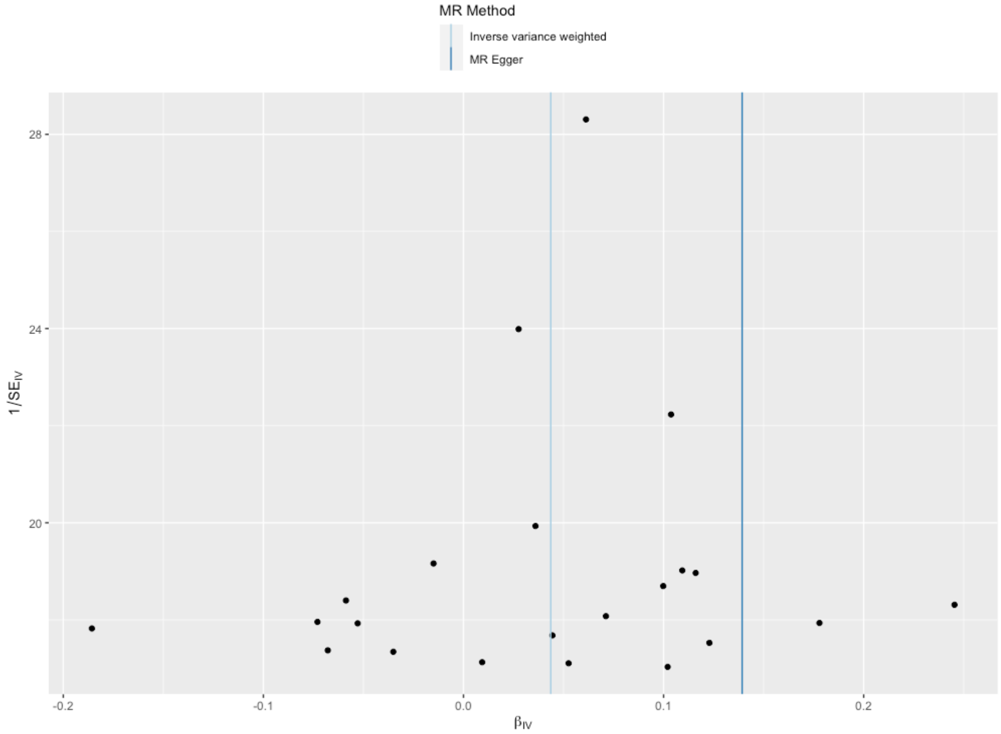

Supplementary Table S1 SNPs site information for Mendelian randomization analysis was performed

SNP	chr	pos	effect_allele	other_allele	se	beta	pval	samplesize
rs840281	3	107691697	G	A	0.0031689	0.000505912	8.73E-01	330759
rs13002946	2	100801959	A	T	0.00253412	0.00441682	8.13E-02	330759
rs2240857	7	8010634	G	T	0.00321021	-0.00691366	3.13E-02	330759
rs1880692	11	80338069	G	A	0.00224944	0.00264995	2.39E-01	330759
rs2806047	14	73532676	G	A	0.00234443	-0.00775969	9.33E-04	330759
rs619466	18	53198836	G	A	0.00385346	-0.0122949	1.42E-03	330759
rs6944796	7	104505787	T	C	0.00276794	0.00516537	6.20E-02	330759
rs1455351	2	199492201	G	A	0.00227371	0.0021554	3.43E-01	330759
rs13019832	2	60710571	A	G	0.00227529	0.005007	2.78E-02	330759
rs10515086	5	67781021	T	C	0.00297914	-0.00213597	4.73E-01	330759
rs4662381	2	145319098	A	G	0.0102353	0.00293707	7.74E-01	330759
rs1889588	1	44012923	T	C	0.00250013	-0.0043445	8.23E-02	330759
rs3741368	11	66083782	A	G	0.00225149	-0.00295103	1.90E-01	330759
rs62366190	5	60483935	G	C	0.00236249	-0.000543358	8.18E-01	330759
rs12553324	9	23347865	G	C	0.00227695	0.000691369	7.61E-01	330759
rs9375188	6	98555272	T	C	0.00224573	0.00389181	8.31E-02	330759
rs77875796	17	44051612	G	A	0.00269731	-0.0121191	7.02E-06	330759
rs55747393	2	236832758	T	C	0.00234277	0.00142509	5.43E-01	330759
rs2726036	16	28347140	C	A	0.00229034	0.00247526	2.80E-01	330759
rs5576953	2	21263408	T	A	0.0024412	-0.00203066	4.06E-0	330759

6		4			7		1	
rs4886031	13	58372213	C	T	0.00249783	0.00196256	4.32E-01	330759
rs1627527	19	3555498	A	G	0.00226097	0.0020314	3.69E-01	330759
rs7634084	3	49949834	T	A	0.0022462	-0.00129513	5.64E-01	330759
rs11654986	17	57206124	G	A	0.00230033	0.00295971	1.98E-01	330759
rs9964724	18	35159124	T	C	0.002411	0.00555969	2.11E-02	330759
rs12210020	6	152237468	G	A	0.00258428	0.00537377	3.76E-02	330759
rs448809	5	88027149	T	G	0.0022807	-0.00150776	5.09E-01	330759
rs68119843	9	124611516	T	A	0.00226801	-0.000234725	9.18E-01	330759
rs9530530	13	76809200	G	C	0.00284597	-0.00282591	3.21E-01	330759
